# The Extract of *Angelica sinensis* Inhibits Hypoxia–Reoxygenation and Copper-Induced Oxidative Lesions and Apoptosis in Branchiae and Red Blood Corpuscles of Fish

**DOI:** 10.3390/vetsci11010001

**Published:** 2023-12-19

**Authors:** Jiao Long, Pengyan Yang, Yihua Liu, Xiaoru Liu, Huatao Li, Xiaoyu Su, Ting Zhang, Jing Xu, Gangfu Chen, Jun Jiang

**Affiliations:** 1 Key Laboratory of Sichuan Province for Conservation and Utilization of Fishes Resources in the Upper Reaches of the Yangtze River, College of Life Sciences, Neijiang Normal University, Neijiang 641100, China; longjiao1005@163.com (J.L.); 18090203417@163.com (P.Y.); liuyihua0814@163.com (Y.L.); 13056566137@163.com (X.L.); s13551569148@163.com (X.S.); 15282750340@163.com (T.Z.); jingxusc@163.com (J.X.); fanyahr@163.com (G.C.); 2 College of Animal Science and Technology, Sichuan Agricultural University, Chengdu 611130, China; jjun@sicau.edu.cn

**Keywords:** *Angelica sinensis*, branchia, red blood corpuscle, Cu, hypoxia–reoxygenation, oxidative lesion, apoptosis

## Abstract

**Simple Summary:**

In aquaculture systems, copper sulfate is regularly used to control algal growth, parasitic infections and Saprolegniasis. In shallow freshwater bodies, the natural oxygen level can vary widely over the course of a day from low levels during the night to sometimes heavy oversaturation during the day. Thus, aquatic organisms may suffer from exposure to excessive concentrations of copper sulfate and hypoxia–reoxygenation stress. However, the way of suppressing copper sulfate and hypoxia–reoxygenation stress is not fully understood in fish. This research provides information that dietary *Angelica sinensis* extract improved hypoxia tolerance and inhibited copper sulfate and hypoxia–reoxygenation-triggered oxidative lesions and apoptosis in branchiae and red blood corpuscles in carp (*Cyprinus carpio* var. Jian). The findings may pave the way for considering *Angelica sinensis* extract as a potential natural mitigator against stress induced by copper sulfate and hypoxia–reoxygenation in fish.

**Abstract:**

The study explored the effects of *Angelica sinensis* extract (AsE) on oxidative lesions and apoptosis in branchiae and red blood corpuscles in hypoxia–reoxygenation (HR) and Cu-treated carp (*Cyprinus carpio* var. Jian). After feeding trial for 30 days, the carp were exposed to HR and CuSO_4_. The results indicated that dietary AsE increased the durative time, decreased the oxygen consumption rate, suppressed ROS generation and cellular component oxidation, decreased enzymatic antioxidant activity and reduced glutathione (GSH) levels in red blood corpuscles and branchiae in carp under hypoxia. Moreover, dietary AsE avoided the loss of Na^+^,K^+^-ATPase, metabolic and antioxidant enzyme activities, ROS generation and cellular component oxidation, as well as the increase in caspase-8, 9, and 3 activities in the branchiae of the carp and inhibited ROS generation. It furthermore avoided the loss of Na^+^,K^+^-ATPase and metabolic enzyme activities, the decrease in GSH levels and hemoglobin content, the increase in the activities of caspase-8, 9, and 3 and the increase in the levels of cytochrome c and phosphatidylserine exposure in the red blood corpuscles of Cu-exposed carp. The present results suggested that dietary AsE improved hypoxia tolerance and inhibited HR or Cu-triggered oxidative lesions and apoptosis. Therefore, AsE can be utilized as a natural inhibitor of Cu and HR stress in fish.

## 1. Introduction

Being a sort of disinfectant, copper sulfate (CuSO_4_) is widely used in the control of parasite infections as well as algae growth in systems of aquaculture sustainability [1,2]. The branchia plays a pivotal role in respiratory functions, specifically in managing the discharge of water containing nitrogen and overseeing gaseous exchanges in fish [3]. However, researchers show the primary accretion of Cu in the branchiae of fish [4]. Moreover, Cu stimulates oxidative lesions as well as apoptosis in fish branchia cells [5,6]. Red blood cells significantly contribute to the movement of both CO_2_ and O_2_ throughout the circulatory system of fish [7,8]. However, it has been reported that fish can absorb Cu via fish branchiae, body surface and digestive tract [9] and significantly increase the content of Cu in the blood [1,10]. Cu leads to oxidative stress and apoptosis in the hemocyte of white shrimp [11]. As reported, Cu exposure will reduce respiratory function in common carp [12]. Therefore, CuSO_4_ may cause oxidative lesions and apoptosis of branchiae and red blood corpuscles, which may lead to a weakness in the respiratory function of fish. However, little information on how Cu affects red blood corpuscle oxidative lesions and apoptosis in fish in vivo has been found.

In aquaculture, fish frequently experience hypoxia–reoxygenation conditions [13]. Concurrently, the presence of Cu can swiftly promote the production of reactive oxygen species (ROS) [14], leading to potential oxidative damage or apoptosis in the fish’s branchiae and red blood cells [13,15,16]. Several enzymatic antioxidants, such as superoxide dismutase (SOD), catalase (CAT) and glutathione peroxidase (GPx), act to neutralize ROS and mitigate their harmful effects [17]. However, studies suggest that the combination of hypoxia–reoxygenation and CuSO_4_ can modify their antioxidant defense strategies [13,18] and negatively affect the fish’s immunity and overall functionality [19,20,21]. Given these insights, there is a pressing need to deepen our understanding of protective measures for the branchiae and red blood cells against the oxidative damage and apoptosis induced by CuSO_4_ and hypoxia–reoxygenation.

*Angelica sinensis* is a precious herb for medicine as well as a well-known food material with a lot of pharmacological functions [22]. Based on research findings, the inclusion of *Angelica sinensis* extract (AsE) in fish diets appears to hinder ROS and lipid oxidation. Dietary AsE enhances the capacity of enzymatic antioxidants in the hepatopancreas and intestines of fish [23,24,25]. Furthermore, in China, the production of Angelica sinensis has reached 80,000 tons [26,27]. The utilization of a large amount of Angelica by-products generated during harvesting and processing is very limited [28]. Therefore, a reasonable hypothesis that AsE can lessen oxidative lesions or apoptosis caused by CuSO_4_ and hypoxia–reoxygenation in fish branchiae and red blood corpuscles exists. Limited studies have been conducted to elucidate the specific influence of AsE on fish’s branchiae and red blood cells in vivo, particularly after exposure to CuSO_4_ combined with hypoxia–reoxygenation scenarios.

This investigation delved into how AsE impacts apoptosis and oxidative damage in the branchiae and red blood cells of carp (*Cyprinus carpio* var. Jian) exposed to CuSO_4_ and conditions of hypoxia–reoxygenation. The primary objective was to discern the modulatory role of AsE on respiratory processes in fish when subjected to CuSO_4_ and hypoxia–reoxygenation treatments. The findings may pave the way for considering AsE as a potential natural mitigator against stress induced by CuSO_4_ and hypoxia–reoxygenation in fish.

## 2. Materials and Methods

### 2.1. Chemical Reagent

The Chengdu Kelong Chemical Reagent Factory, located in Chengdu, Sichuan, China, supplied the analytical-grade ethyl acetate, ethyl ether and methanol. The analytical-grade copper sulfate pentahydrate (CuSO_4_·5H_2_O) was sourced from the Shanghai Chemical Reagent Factory located in Putuo District, Shanghai, China. Ligustilide, with a purity of at least 98%, came from Chengdu Herbpurify Co., LTD located in Chengdu, Sichuan, China. Both quercetin and ferulic acid, with a purity level of not less than 99%, were acquired from Shanghai EkearBiotech. Co., Ltd. located in Fengxian District, Shanghai, China. Sigma-Aldrich Co., LLC, based in St. Louis, MO, USA, provided catechin, kaempferol and heparin sodium, each boasting a purity of 99% or higher. Every other chemical used for this research met the analytical-grade criteria.

### 2.2. AsE Preparation and Composition Analyses

**Figure 1:**
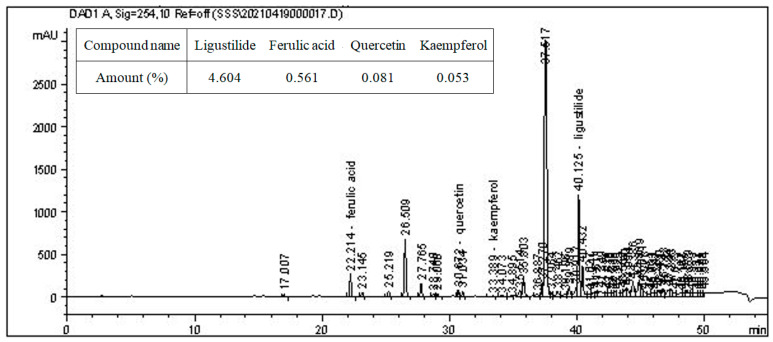
The composition analyses of ethyl acetate extract (EAE) of *Angelica sinensis* by high-performance liquid chromatography (HPLC). This experiment was repeated three times with similar results achieved.

The root of *Angelica sinensis* was sourced from the Chengdu pharmaceuticals market, located in Chengdu, Sichuan, China. Based on guidelines by the report [29], we conducted botanical identification and prepared AsE. Prior studies indicated that the ethyl acetate extract from *Angelica sinensis* (EAE) exhibited significant protective attributes against lipid oxidation in aquatic feed and amplified carp growth performance [29]. Consequently, we utilized EAE in the subsequent experiments. Li et al., 2020 described the composition analysis methodology of EAE using high-performance liquid chromatography (HPLC) [30], which we slightly modified. By dissolving EAE in methanol of a chromatography grade, we prepared a concentrated solution. Before employing a 0.22 μm pinhole germ filter, this solution underwent HPLC to quantitatively analyze constituents like ligustilide, ferulic acid, catechin, quercetin, and kaempferol. Our HPLC instrument (Agilent 1100 HPLC, Wilmington, DE, USA) incorporated an Agilent Zorbax SB C18 column (250 mm ⊆ 4.6 mm i.d., 5 μm particle size). We used the solvents (A) 0.5% formic acid in water and (B) pure methanol, adjusting the A:B gradient from 95:5 to 5:95 over 45 min. The flow rate of the mobile stage was consistently maintained at 1.0 mL min^−1^, and peaks were detected at 254 nm. We prepared three replicates for every sample. Figure 1 delineates the contents of the mentioned EAE components.

### 2.3. Diets and Feeding Trial

Earlier research revealed that fish attained a maximum weight gain and feed efficiency when consuming a diet comprising 4.0 g of EAE per kg [29]. Based on this observation, a concentration of 4.0 g of EAE per kg feed was chosen for the subsequent trials. We devised two experimental diets: the foundational one and another augmented with EAE, with the formulation process described in prior work [29]. The foundational diet had components of 5.63% crude lipids and 34.17% crude proteins. Introducing EAE achieved the desired concentration of 4.0 g of EAE per kg, with a concurrent decrease in Soybean oil to balance out the EAE increment. The diet formulations are presented in Table 1. The feeding protocol adopted mirrored the methodology from Li et al., 2019 [29]. Following a 15-day acclimatization period in lab settings, a total of 240 fish, each averaging a weight of 9.71 ± 0.10 g, were distributed at random into two categories: the baseline group and the EAE group. Each group contained 6 fish tanks with 20 fish in each tank. These groups were administered the foundational and EAE diets over a span of 30 days, respectively.

### 2.4. Cu Exposure

Upon the conclusion of the feeding experiment, we exposed 60 fish in 3 tanks from both the baseline and EAE groups to water containing 3.125 mg of CuSO_4_·5H_2_O L^−1^ over a period of four days, respectively, adhering to methods documented in earlier studies [25,30]. Conversely, another 60 fish in another 3 tanks,, originating from both the baseline and EAE groups were immersed in unpolluted water, respectively. For each exposure condition, we maintained 3 separate tanks, with each tank housing a total of 20 fish. These steps were executed while maintaining conditions consistent with the feeding experiment.

Upon the completion of Cu exposure, 10 fish in each tank received an anesthetic treatment involving ethyl carbamate and were subsequently placed in an ice-cooled environment. Researchers harvested blood samples from the 10 fish through caudal venipuncture with syringes treated with heparin. These blood samples underwent a centrifugal process at 1000× *g* and 4 °C for a duration of 3 min within an hour of collection. Hematological indices were assessed from the separated red blood corpuscles and plasma. The methodologies outlined in an earlier study [8] facilitated the determination of the red blood corpuscle count (RBC), haematocrit (Hct) and haemoglobin concentration (HbC) in the samples. Based on these results, computations were made for the mean corpuscular haemoglobin content (MCH) (HbC divided by RBC), mean corpuscular volume (MCV) (Hct divided by RBC), and mean corpuscular haemoglobin concentration (MCHC) (HbC divided by Hct). Subsequently, specific analyses were conducted on the isolated red blood corpuscles, examining parameters like phosphatidylserine (PS) exposure (a biomarker of apoptosis), cytochrome c, hydroxyl radicals (·OH), met-hemoglobin (Met-Hb), reduced glutathione (GSH) levels, and specific enzymatic activities, such as those of caspase-3, caspase-8, caspase-9, Na^+^,K^+^-ATPase, lactate dehydrogenase (LDH), glutamate-oxaloacetate transaminase (GOT) and glutamate-pyruvate transaminase (GPT). Branchial samples were extracted and preserved at −80 °C to later evaluate the content of hydrogen peroxide (H_2_O_2_) and malondialdehyde (MDA), along with the activities of enzymes like SOD, CAT, GPx, Na^+^,K^+^-ATPase, GPT, GOT, caspase-3, caspase-8, and caspase-9.

### 2.5. Hypoxia–Reoxygenation Assays

Upon concluding the Cu exposure trial, researchers harvested 10 fish from each tank to record their weight (W, g) and assess their hypoxia endurance. To measure the oxygen consumption rate (OCR, mg g^−1^ h^−1^), we adopted the method from Li et al., 2020 [13], introducing minor adjustments. In essence, we stabilized 40 L of fresh tap water to an ambient temperature (22 °C) in the tanks, ensuring it reached a saturated dissolved oxygen (DO) level over 24 h via an aerator (SB-748, Shanghai, China). Ahead of our measurements, a volume (V, L) equivalent to 20 times the weight of the saturated DO water was meticulously moved to a specialized bottle (polyethylene terephthalate, 4.5 L capacity). This bottle then housed ten fish. Excess air within was expelled through the gentle compression of the bottle. Using the Leici JPBJ-608 analyzer (Shanghai, China), we gauged the initial DO (IDO, mg L^−1^). The bottle was promptly sealed, and the clock began, recording the start time (ST). Except for the top of the bottle, all sides of the bottle were surrounded by opaque yellow kraft paper. Observers needed to maintain a distance of 20–30 cm from the bottle and could not come into contact with it. They could only observe the situation of the fish through the window on the kraft paper. On observing an imbalance in 8 out of the 10 fish, we halted the timer, recording the end time and swiftly assessed the final DO (FDO, mg L^−1^). The fish were then quickly shifted to a saturated DO environment for a 2 h reoxygenation phase. We used the elapsed time as the duration (DT, h). The criterion for fish imbalance is when they lie flat on the bottom of the bottle and cannot stand upright. The OCR was determined for each hour and per gram of fish weight. We replicated this procedure thrice for every treatment condition.

$$OCR=\frac{\left(IDO-FDO\right)\times V}{DT\times W}$$

Following the hypoxia–reoxygenation process, the investigators harvested blood samples from each fish in the tanks using caudal puncture techniques. From these samples, red blood cells were extracted to assess the levels of superoxide anions (O_2_^−^), H_2_O_2_, MDA and Met-Hb, alongside the activities of the CAT, SOD and GPx enzymes. Fish gills were carefully extracted and preserved at a temperature of −80 °C. The subsequent analysis targeted the levels of O_2_^−^, ·OH, MDA, protein carbonyls (PC) and GSH, in addition to the enzymatic activities of glutathione reductase (GR) and glutathione-S-transferase (GST).

The procedures detailed previously received the green light from Neijiang Normal University’s Institutional Animal Care and Use Committee. These practices align with the ethical standards set by the Chinese Institute of Chemical Biology’s Institutional Ethics Committee.

### 2.6. Biochemical Analysis

For the assessment of GPT and GOT activities, we referred to the protocols from Huang et al., 2019 [31]. The methods presented by Chen et al., 2009 [32] guided the evaluations for LDH and Na^+^,K^+^-ATPase activities. The concentrations of O_2_^−^ and MDA were gauged in accordance with Lin et al., 2011 [33]. Assays for SOD, CAT and GPx were conducted using methodologies delineated by Jiang et al., 2009 [34]. Parameters like Met-Hb, PC, GSH and H_2_O_2_ in red blood corpuscles were determined following Li et al., 2016 [35]. Li et al., 2019 [23] provided insights into measuring ·OH levels and GR and GST activities. To quantify the protein content, we used the method detailed in a previously reported study [36]. Lastly, the Darbkin 1946 [37] approach was applied for Hb concentration determination.

### 2.7. Apoptosis and Caspases Measurement

Utilizing the Annexin V-FITC Apoptosis Detection Kit from Beyotime (Nantong, China), we evaluated the exposure of PS in fish red blood corpuscles, drawing from established protocols [17]. For the assessment of caspase 3, 8, and 9 activities, specific detection kits, also sourced from Beyotime, were deployed. These kits measure the conversion of Ac-DEVD-pNA (for caspase-3), Ac-IETD-pNA (for caspase-8) and Ac-LEHD-pNA (for caspase-9) to pNA, following methodologies described in prior research [16]. We employed the Microplate reader from Thermo (Waltham, MA, USA) to capture caspase activities in the samples, gauging absorbance at 405 nm.

### 2.8. Cytochrome c Measurement

Utilizing a specialized extraction kit sourced from Beyotime (Nantong, China), we extracted the mitochondria and cytosolic proteins from fish red blood corpuscles. To quantify the cytochrome c released from the cytosol mitochondria, we employed an ELISA kit from ElabScience (Wuhan, China), drawing from methodologies highlighted in the report of Li et al., 2016 [16]. The absorbance of the samples was gauged using a Microplate reader, set at a wavelength of 450 nm.

### 2.9. Statistical Analysis

Data are presented as the mean complemented by the standard deviation (S.D.). To evaluate the results, both a two-way ANOVA and *t*-test were utilized, as referenced in the reports [18,38]. Notable differences in the results were identified using Duncan’s multiple range tests, as cited in the report [39]. For statistical analysis, the software tool employed was SPSS 15.0, available for Windows (originating from IBM, Amonk, New York, NY, USA), with a chosen significance threshold set at 95% (α = 0.05).

## 3. Results

### 3.1. How Dietary EAE Influences Hypoxia Tolerance and the Biochemical Parameters in Branchiae and Red Blood Corpuscles of Hypoxia–Reoxygenation-Treated Carp

When juxtaposed with the control group, the carp that consumed a diet inclusive of EAE exhibited an enhanced DT and a reduced OCR under hypoxic conditions (*p* < 0.05), as presented in Table 2.

Under the influence of dietary EAE intake, the branchial levels of O_2_^−^, ·OH, MDA and PC significantly diminished, while there was a notable upswing in the GSH level and the activities of GR and GST (*p* < 0.05), as depicted in Table 3. In contrast, the exposure to hypoxia–reoxygenation led to an elevation in the levels of O_2_^·−^, ·OH, MDA and PC and the activity of GR. It also contributed to a decline in the GSH level and GST activities in branchiae (*p* < 0.05). However, the inclusion of EAE in the diet led to noticeable alterations; it lowered the O_2_^−^, ·OH and MDA concentrations while amplifying the GSH level and the activities of GR and GST under hypoxia–reoxygenation scenarios (Table 3).

Relative to the control, the carp fed with EAE demonstrated a notable reduction in Met-Hb and MDA concentrations while showing heightened CAT activities within their red blood corpuscles (*p* < 0.05), as illustrated in Table 4. In contrast, the exposure to the hypoxia–reoxygenation process led to a pronounced elevation in the levels of Met-Hb, O_2_^−^, ·OH and MDA, an amplified SOD activity and a decline in the activities of both GPx and CAT in the red blood corpuscles of the carp (*p* < 0.05), as detailed in Table 4. Intriguingly, the EAE-infused diet counteracted these alterations in the carp subjected to the hypoxia–reoxygenation condition (*p* < 0.05), as presented in Table 4.

### 3.2. How Dietary EAE Influences Hypoxia Tolerance and the Biochemical Parameters in Branchiae in Cu-Treated Carp

In comparison with the control group, the DT was decreased and OCR was enhanced in the Cu treatment group under hypoxia (*p* < 0.05) (Table 2). Nonetheless, dietary EAE reversed the parameters above in the Cu treatment group (*p* < 0.05) (Table 2).

**Figure 2:**
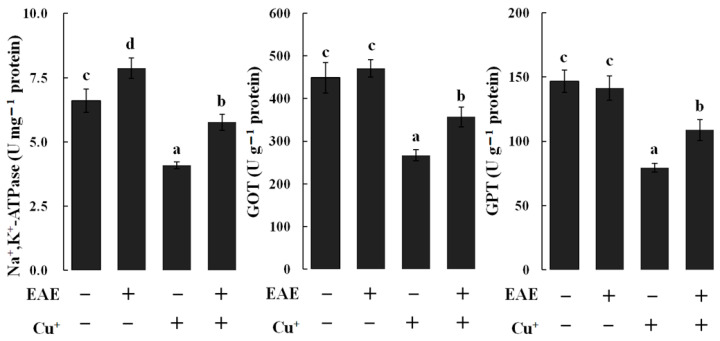
The activities of Na^+^,K^+^-ATPase, glutamate-oxaloacetate transaminase (GOT) and glutamate-pyruvate transaminase (GPT) in branchiae of carp fed diets containing an ethyl acetate extract of *Angelica sinensis* (EAE). The data represent the means ± S.D. of 3 replicates, with 10 fish in each replicate. The values that do not share a superscript are significantly different (*p* < 0.05).

When set against the control group, the Cu-exposed group’s branchiae displayed notably diminished activity levels for Na^+^,K^+^-ATPase, GPT and GOT (*p* < 0.05), as depicted in Figure 2. Yet, this trend was counteracted when the fish consumed a diet infused with EAE, indicating its potential protective effects against Cu exposure (*p* < 0.05), as visualized in Figure 2. Additionally, the branchiae from the EAE-fed group alone manifested an uptick in Na^+^,K^+^-ATPase activity (*p* < 0.05), as highlighted in Figure 2.

**Figure 3:**
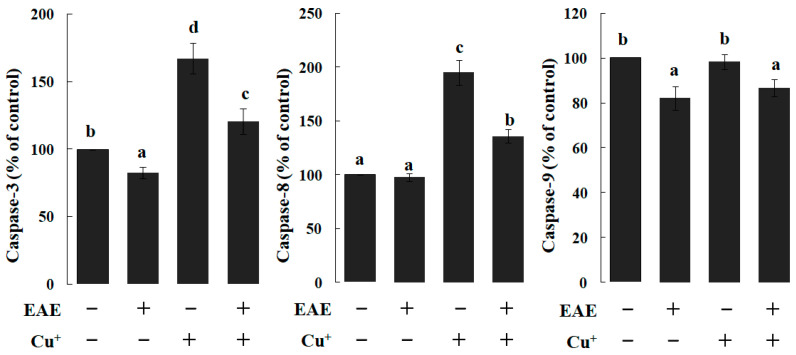
The activities of caspase-3, caspase-8 and caspase-9 in branchiae of carp fed diets containing an ethyl acetate extract of *Angelica sinensis* (EAE). The data represent the means ± S.D. of 3 replicates, with 10 fish in each replicate. The values that do not share a superscript are significantly different (*p* < 0.05).

Within the branchiae of the fish from the dietary EAE group, the activity of caspase 3 and 9 were notably reduced when benchmarked against their control counterparts. Meanwhile, caspases 3 and 8 exhibited a discernible surge in activity within the Cu-exposed fish’s branchiae (*p* < 0.05), as depicted in Figure 3. However, the introduction of dietary EAE appeared to counteract these elevated activities in the Cu-exposed carp’s branchiae (*p* < 0.05) (Refer to Figure 3).

Relative to the control group, the MDA concentration in the branchiae of the dietary EAE group showed a noticeable reduction, while the activities of SOD, CAT and GPx trended upwards. In the branchiae of the fish exposed to Cu, there was a noticeable elevation in H_2_O_2_ and MDA concentrations and an enhanced activity of GPx. Concurrently, the SOD and CAT activities witnessed a decline (*p* < 0.05), as outlined in Table 5. However, supplementing with dietary EAE resulted in a prominent drop in H_2_O_2_ and MDA concentrations and bolstered the activities of CAT, SOD and GPx within the branchiae of the Cu-exposed fish (*p* < 0.05), as detailed in Table 5.

### 3.3. How Dietary EAE Influences the Hematochemical and Hematological Index in Cu-Treated Carp

**Figure 4:**
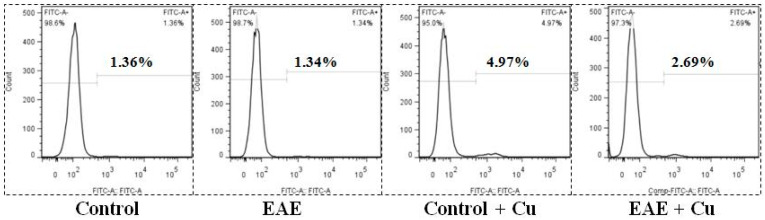
The levels of annexin binding in red blood corpuscles of carp fed diets containing ethyl an acetate extract of *Angelica sinensis* (EAE). The experiment was repeated three times with similar results achieved.

**Figure 5:**
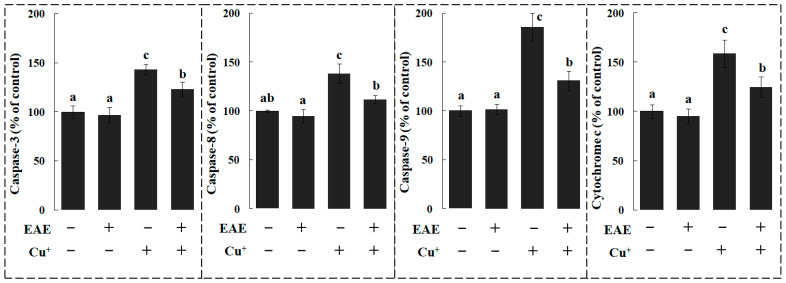
The activities of caspase-3, caspase-8 and caspase-9 and the level of cytochrome c in red blood corpuscles of carp fed diets containing an ethyl acetate extract of *Angelica sinensis* (EAE). The data represent the means ± S.D. of 3 replicates, with 10 fish in each replicate. The values that do not share a superscript are significantly different (*p* < 0.05).

Relative to the control group, Cu exposure led to a significant rise in annexin binding, cytochrome c concentrations, and the activities of caspases 3, 8, and 9 within the red blood corpuscles of the carp, as illustrated in Figure 4 and Figure 5. Conversely, the introduction of dietary EAE demonstrated its efficacy by mitigating these changes in the red blood corpuscles of the carp subjected to a Cu treatment, as depicted in Figure 4 and Figure 5.

Relative to the control group, the introduction of dietary EAE was observed to elevate the levels of Hct, RBC and HbC in the carp. On the other hand, the exposure to Cu led to a discernible reduction in the carp’s blood metrics of Hct, RBC, HbC and MCH, as detailed in Table 6. Yet, the supplementation of EAE counteracted the diminishing effects exerted by Cu on these specific parameters, as reflected in Table 6.

Upon exposure to Cu, a significant surge in the ·OH level was observed in the red blood corpuscles of the carp, as detailed in Table 7. Yet, the inclusion of dietary EAE resulted in a notable reduction in both the ·OH and Met-Hb levels in the carp’s red blood corpuscles exposed to Cu (*p* < 0.05). Moreover, even without Cu exposure, dietary EAE contributed to a decline in these two metrics (*p* < 0.05) (Table 7). Compared to the control set, the carp’s red blood corpuscles under dietary EAE treatment demonstrated elevated levels of GSH and increased activities of Na^+^,K^+^-ATPase, LDH and GPT. In the Cu-exposed group, there was a noticeable drop in the GSH levels and the activities of Na^+^,K^+^-ATPase, LDH, GPT and GOT. Yet, these declines were mitigated with the inclusion of dietary EAE in the Cu-exposed carp (*p* < 0.05) (Table 7).

## 4. Discussion

### 4.1. Dietary EAE Improved Hypoxia Tolerance and Suppressed Hypoxia–Reoxygenation-Induecd Oxidative Lesions in Branchiae and Red Blood Corpuscles of Fish

Our earlier work revealed that supplementing carp with dietary EAE for 30 days notably enhanced their growth [29]. In this study, we found that dietary EAE supplementation improved the DT while reducing the OCR in carps exposed to hypoxia. Such outcomes suggest a heightened hypoxia tolerance in fish due to EAE. Consistently, it aligns with the prior literature suggesting a link between fish growth and their ability to withstand hypoxic conditions [40]. The primary roles of branchiae involve nitrogenous waste elimination and facilitating gas exchange essential for fish respiration [3]. Red blood corpuscles, on the other hand, primarily facilitate O_2_ transport and orchestrate CO_2_ production during respiration [41]. However, Hb can autonomously undergo oxidation, producing ROS and Met-Hb that cannot bind O_2_ [42]. Reactive oxygen species such as O_2_^·−^, H_2_O_2_ and ·OH can potentially oxidize cellular components, leading to the formation of MDA and PC, which can subsequently inactivate essential cellular enzymes [17]. Notably, fish branchiae possess abundant red blood corpuscles [3], suggesting that branchial functions might be influenced by their ROS levels. In this context, we observed that EAE supplementation mitigated the rise in oxidative markers in both branchiae and red blood corpuscles of carps subjected to hypoxia–reoxygenation. Such findings emphasize that hypoxia–reoxygenation can induce ROS production and oxidative damage in these cells, echoing previous studies [13]. Furthermore, our observations highlight that dietary EAE can counteract such oxidative challenges, reminiscent of findings reported in fish digestive organs [24]. Hence, our findings provide evidence that hypoxia–reoxygenation can promote oxidative stress in fish branchiae and red blood corpuscles, but dietary EAE can offer protective effects against such oxidative damages.

Antioxidant enzymes present in fish cells play a pivotal role in neutralizing intracellular ROS and preventing oxidative damage to cellular components [43]. Our investigations underscored that dietary EAE counteracted the diminished activity of GPx and CAT in the carp’s red blood corpuscles upon exposure to hypoxia–reoxygenation. This observation resonates with previous findings, indicating enhanced activity of antioxidant enzymes in the hepatopancreas and intestines of fish supplemented with AsE [24]. Serving as a primary non-enzymatic antioxidant, GSH directly neutralizes intracellular ROS [44]. Furthermore, the enzymatic conversion of oxidized GSH (GSSG) back to its reduced form, aided by GR, ensures high intracellular GSH levels [29]. GST assisted in conjugating lipid peroxides’ cleavage products by taking GSH as the substrate [45]. In the current research, dietary EAE assisted in enhancing the above indicator and improved the hypoxia–reoxygenation- caused decrease in GSH level and GST activity in carp branchiae, which well supported a report on the hepatopancreas and intestines of fish [24]. As revealed, dietary EAE could quench hypoxia–reoxygenation-caused oxidative lesions by elevating fish branchiae or red blood corpuscles’ enzymatic antioxidant activity and non-enzymatic antioxidant levels.

### 4.2. Dietary EAE Suppressed Cu-Caused Oxidative Lesions and Apoptosis in Branchiae of Fish

Studies have highlighted the branchiae as the primary organ susceptible to Cu toxicity in fish [1]. Na^+^,K^+^-ATPase plays a vital role in maintaining ion electrochemical gradients across fish branchial plasma membranes [46]. Disruptions to the activity of this enzyme could compromise both respiratory and ionoregulatory mechanisms [1]. This investigation revealed that dietary EAE mitigated the adverse impact of Cu on hypoxia tolerance and this particular enzyme in carp branchiae. When considering protein metabolism, GPT and GOT emerge as crucial enzymes, and their activity levels provide insights into amino acid utilization within fish organs [25]. Our data indicate that dietary EAE countered the reduction in the GPT and GOT activities triggered by Cu in the carp’s branchiae. This observation aligns with outcomes observed in trichlorfon-exposed fish [8]. Cu exposure seemed to compromise specific functionalities in fish branchiae, but the detrimental effects were attenuated with dietary EAE supplementation. Furthermore, the study identified a reduction in H_2_O_2_ and MDA levels in carp branchiae when they were provided with dietary EAE, despite Cu exposure. Drawing from the above, Cu’s presence appears to promote ROS generation, leading to the oxidation of cellular components in the branchiae, a notion reinforced by the report [18]. The overarching conclusion from these findings suggests that Cu induces oxidative damage in fish branchiae, but dietary EAE supplementation offers protection, mirroring outcomes observed in fish digestive systems [25].

Luzio et al., 2013 [47] have documented that Cu triggers apoptosis in fish branchiae through both intrinsic pathways implicating caspase-9 and 3 and extrinsic pathways implicating caspase-8 and 3. In our present investigation, the administration of dietary EAE was observed to counteract the upregulation of caspases 3 and 8 induced by Cu. This suggests that EAE can act as a defensive mechanism against apoptosis in fish branchiae triggered by Cu, which aligns with previous findings in carp exposed to trichlorfon [8]. The involvement of ROS in facilitating Cu-induced apoptosis in aquatic organisms, particularly in fish branchiae or hemocytes, is noteworthy [11,15]. Interestingly, our research emphasizes that EAE supplementation amplifies the activity of antioxidant enzymes like SOD, GPx, and CAT. It also offers a protective shield against Cu-induced reductions in CAT and SOD enzymatic activities in carp branchiae. This mirrors previous observations where Cu was identified to diminish the activities of enzymatic antioxidants in branchiae [18], while dietary AsE was found to thwart this decrease in fish hepatopancreas and intestines [25]. Collectively, our results insinuate that dietary EAE potentially combats oxidative damage and apoptosis provoked by Cu in fish branchiae, chiefly by bolstering their enzymatic antioxidant defenses. This concurs with patterns observed in trichlorfon-exposed fish [8]. The exact mechanisms through which AsE influences these observed changes in Cu-exposed fish branchiae warrant further investigation.

### 4.3. Dietary EAE Suppressed Cu-Caused Oxidative Lesions as Well as Apoptosis in Fish Red Blood Corpuscles

Na^+^,K^+^-ATPase plays an essential role in preserving the cytoplasmic ionic environment, which is pivotal to preventing colloidal osmotic lysis in red blood corpuscles [41]. LDH, an indispensable enzyme in anaerobic glycolysis, facilitates energy production [48], which in turn safeguards the function of Na^+^,K^+^-ATPase within these cells [41]. Our investigation reveals that carp red blood corpuscles benefit from dietary EAE supplementation, which counteracts the detrimental effects of Cu on the activities of Na^+^,K^+^-ATPase and LDH. These observations are consistent with previous findings in trichlorfon-exposed carp [8]. According to Tiihonenk and Nikinmaa 1991 [49], carp’s red blood corpuscles can harness amino acids as an energy substrate. GPT and GOT are instrumental in channeling amino acids into the tricarboxylic acid cycle through deamination, facilitating energy production [50,51]. Our study highlights that dietary EAE alleviates the adverse effects of Cu on GPT and GOT activities within these cells. The data align with earlier research focused on carp branchiae. Additionally, dietary EAE moderates the impact of Cu, as evident by attenuating a reduction in hematological indices like Hct, RBC, HbC and MCH. This suggests Cu’s propensity to diminish red blood corpuscle counts and hemoglobin content, whereas dietary EAE appears to bolster these hematological markers in fish blood, resonating with findings in rainbow trout [52] and trichlorfon-exposed carp [8]. Limited studies explore the effects of Cu on GOT and GPT in fish’s red blood corpuscles or the potential role of AsE in influencing hematological indices in Cu-exposed fish. In summary, our findings underscore Cu’s adverse impacts on fish red blood corpuscles, predominantly through metabolic disruption and compromised O_2_ transportation. Conversely, dietary EAE shows potential in mitigating these Cu-induced challenges.

The interplay between Cu’s impact on the functional attributes and the ROS status of fish’s red blood corpuscles merits attention. Our study highlights that when carp are exposed to Cu, dietary EAE successfully mitigates the rise in the levels of ·OH and Met-Hb within their red blood corpuscles. This underscores Cu’s propensity to induce ROS generation and, conversely, EAE’s capability to counteract this induction. Such observations are congruent with previously documented findings that Cu exposure has been shown to amplify ROS production in white shrimp’s hemocytes [11], while dietary AsE enhances the capacity to neutralize ROS in the digestive organs of fish exposed to Cu [25]. An overarching insight from our research indicates that Cu does more than merely boost ROS production—it also precipitates a decline in functional efficiency, suggesting that it triggers oxidative damage in fish red blood corpuscles. However, the administration of dietary EAE appears to provide a protective buffer against such Cu-induced oxidative disruptions.

It has been shown that ROS stimulates caspases 8 and 9, respectively, through activating clustered receptors of death (extrinsic pathway) and cytochrome c (intrinsic pathway) that are released, thus activating caspase-3 and successively exposing PS to the surface of fish red blood corpuscles [16]. Here, due to Cu, PS exposure, ROS levels and caspase-8 and 3 activities were enhanced in carp red blood corpuscles, which showed that Cu initiates apoptosis via the extrinsic pathway involved with death receptors and caspase-8 and was in accordance with an article on zebrafish branchia treated with Cu [47]. Moreover, Cu led to an increase in cytochrome c levels and caspase-9 activity in fish red blood corpuscles, which indicated that Cu induces apoptosis through a mitochondria-mediated pathway and supported a report on chicken hepatocytes [53]. Therefore, apoptosis seems to be initiated via two different pathways in fish red blood corpuscles treated with Cu. Not much data can be found on the way that CuSO_4_ influences the apoptotic mechanism in fish red blood corpuscles. However, dietary EAE prevented the increase in PS exposure, cytochrome c levels and caspase-3, caspase-8 and caspase-9 activities in the carp red blood corpuscles caused by Cu. These results confirmed that EAE had the function of protecting red blood corpuscles against apoptosis by decreasing the cytochrome c level and the caspase activity in fish caused by Cu, which well conformed to the report that AsE remarkably decreases keratinocyte apoptosis during catagen in mice [54].

GSH plays a pivotal role as a buffer for sulfhydryl groups (–SH), crucial in ensuring a reduced –SH state within antioxidant enzymes and Hb [41]. In our observations, it became evident that dietary EAE effectively counteracts the decline in the GSH level induced by Cu in fish’s red blood corpuscles. Such findings align consistently with the documented impacts of Cu exposure on the liver, gills, and intestines of fish [18,55], as well as the effects on the hepatopancreas and intestines of carp fed with dietary AsE [24,25]. From a broader perspective, these insights underscore the potential of dietary AsE to alleviate oxidative damage and apoptosis triggered by Cu by bolstering a non-enzymatic antioxidant presence within fish red blood corpuscles.

The protective roles of AsE against oxidative damage and apoptosis in red blood corpuscles might stem from its primary constituents. Notably, ligustilide and ferulic acid have been identified as the principal active components of *Angelica Sinensis* [56]. Within the EAE examined in this study, ligustilide and ferulic acid were present at concentrations of 4.604% and 0.561%, respectively. Intriguingly, the ferulic acid concentration in EAE is notably elevated, approximately 13-fold, compared to its levels (0.040–0.044%) in *Angelica Sinensis* sourced from Sichuan, China [57]. Other research has shed light onto the positive impacts of ligustilide, emphasizing its potential to mitigate lipid and protein oxidation, reduce neuronal apoptosis, and bolster the activities of key enzymes like SOD and CAT within the mouse brain [58,59]. Ferulic acid can be absorbed effectually through the intestinal mucosa in mice [60], easily move into the streams of blood [61], lower the ROS level in rats’ aortas [62] and abrogate the elevation of caspase-3 levels as well as cytochrome c release in the ischemic cortex in rats [63]. However, information regarding the effect of ligustilide and ferulic acid on red blood corpuscles and branchiae is scarce in fish.

## 5. Conclusions

In conclusion, supplementing with AsE positively impacts the hypoxia tolerance of fish, shielding both red blood corpuscles and branchiae from the oxidative damage brought about by hypoxia–reoxygenation. Additionally, AsE counteracts oxidative damage and apoptosis induced by Cu, marked by enhanced Na^+^,K^+^-ATPase, metabolic functions and boosted antioxidant enzyme activities. This elevation in enzymatic activities and non-enzymatic antioxidants, combined with a reduction in caspase activity and cytochrome c release, substantiates AsE’s potential as a natural safeguard against stresses from Cu and hypoxia–reoxygenation in fish.

## Figures and Tables

**Table 1:** Composition and proximate analysis on the basal and ethyl acetate extract of *Angelica sinensis* (EAE) diets.

Ingredients	Basal Diet (%)	EAE Diet (%)	Proximate Analysis ^3^	Content (%)
Fish meal	25.00	25.00	Dry matter	93.48
Soybean meal	30.55	30.55	Crude protein	34.17
Wheat flour	37.17	37.17	Crude lipid	5.63
DL-methionine	0.42	0.42	Crude Ash	7.61
Threonine	0.40	0.40		
Ca(H_2_PO_4_)_2_	1.50	1.50		
Vitamin mixture ^1^	1.00	1.00		
Mineral mixture ^2^	1.00	1.00		
Fish oil	1.16	1.16		
Soybean oil	1.80	1.40		
EAE	0.00	0.40		

^1^ Presented as per kg of vitamin mix below: folic acid (96%), 0.52 g; D-biotin (2%), 5.00 g; meso-inositol (99%), 52.33 g; D-calcium pantothenate (90%), 2.73 g; niacin (99%), 2.82 g; cyanocobalamin (1%), 0.10 g; ascorhyl acetate (93%), 7.16 g; riboflavine (80%), 0.63 g; pyridoxine HCl (81%), 0.92 g; menadione (23%), 0.43 g; thiamin nitrate (90%), 0.11 g; cholecalciferol (500,000 IUg^−1^), 0.48 g; DL-α-tocopherol acetate (50%), 20.00 g; retinyl acetate (500,000 IUg^−1^), 0.80 g. ^2^ Presented as per kg of mineral mix below: CaCO_3_, 897.98 g; KI (4% I), 2.90 g; ZnSO_4_·7H_2_O (23% Zn), 21.64 g; MnSO_4_·H_2_O (32% Mn), 4.09 g; Na_2_SeO_3_·5H_2_O (1% Se), 2.50 g; CuSO_4_·5H_2_O (25% Cu), 1.20 g; FeSO_4_·7H_2_O (20% Fe), 69.70 g. ^3^ Proximate analyses were carried out by the methods of the Association of Official Analytical Chemists (AOAC) [13].

**Table 2:** The effects of dietary ethyl acetate extract of Angelica sinensis (EAE) on the durative time (DT) and oxygen consumption rate (OCR) in juvenile Jian carp under hypoxia.

Item	W (g fish^−1^)	V (L bottle^−1^)	IDO (mg L^−1^)	ST (h:m)	ET (h:m)	DT (h)	FDO (mg L^−1^)	OCR (mg h^−1^g^−1^)
Control	22.03 ± 0.72 ^a^	4.41 ± 0.14 ^a^	8.57 ± 0.04 ^a^	9:34 ± 0:08	11:07 ± 0:04	1.55 ± 0.11 ^b^	0.10 ± 0.01 ^a^	0.109 ± 0.008 ^b^
EAE	21.56 ± 0.90 ^a^	4.31 ± 0.18 ^a^	8.58 ± 0.05 ^a^	9:58 ± 0:08	11:45 ± 0:06	1.78 ± 0.11 ^c^	0.09 ± 0.01 ^a^	0.096 ± 0.006 ^a^
Control + Cu	21.07 ± 0.47 ^a^	4.21 ± 0.09 ^a^	8.56 ± 0.03 ^a^	10:21 ± 0:09	11:40 ± 0:08	1.32 ± 0.07 ^a^	0.11 ± 0.02 ^a^	0.128 ± 0.007 ^c^
EAE + Cu	21.33 ± 0.61 ^a^	4.27 ± 0.12 ^a^	8.57 ± 0.04 ^a^	10:47 ± 0:10	12:18 ± 0:08	1.52 ± 0.09 ^b^	0.10 ± 0.02 ^a^	0.112 ± 0.006 ^b^

W, body weight; V, water volume; IDO, initial dissolved oxygen; ST, start time; ET, end time; FDO, final dissolved oxygen. Values are means ± SD of 3 replicates, with 10 fish in each replicate. Values in the same column with different superscripts are significantly different (*p* < 0.05).

**Table 3:** The levels of superoxide anions (O_2_^−^), hydroxyl radicals (·OH), malondialdehyde (MDA), protein carbonyl (PC) and reduced glutathione (GSH) and the activities of glutathione reductase (GR) and glutathione-S-transferase (GST) in branchiae of carp fed a diet containing an ethyl acetate extract of *Angelica sinensis* (EAE).

Treatment	Control Group	EAE Group	Control + Hypoxia–Reoxygenation	EAE + Hypoxia–Reoxygenation
O_2_^−^ (U g^−1^ protein)	21.19 ± 1.38 ^c^	16.13 ± 0.98 ^a^	30.65 ± 1.82 ^d^	18.29 ± 1.10 ^b^
·OH (U mg^−1^ protein)	17.83 ± 0.73 ^b^	15.59 ± 0.96 ^a^	23.02 ± 1.00 ^d^	19.04 ± 0.78 ^c^
MDA (nmol mg^−1^ protein)	4.93 ± 0.22 ^b^	4.27 ± 0.29 ^a^	6.85 ± 0.50 ^c^	5.02 ± 0.26 ^b^
PC (nmol mg^−1^ protein)	1.36 ± 0.10 ^b^	0.96 ± 0.07 ^a^	1.80 ± 0.11 ^c^	1.47 ± 0.08 ^bc^
GSH (mg g^−1^ protein)	10.51 ± 0.48 ^b^	12.34 ± 0.84 ^c^	8.66 ± 0.63 ^a^	12.00 ± 0.66 ^c^
GR (U g^−1^ protein)	64.63 ± 2.23 ^a^	78.55 ± 6.14 ^b^	76.56 ± 4.77 ^b^	110.30 ± 8.14 ^c^
GST (U mg^−1^ protein)	48.10 ± 3. 54 ^b^	59.66 ± 3.29 ^c^	34.35 ± 2.34 ^a^	46.98 ± 2.94 ^b^

Values are means ± SD of 3 replicates, with 10 fish in each replicate. Values within the same line with different superscripts are significantly different (*p* < 0.05).

**Table 4:** The levels of met-hemoglobin (Met-Hb), superoxide anions (O_2_^−^), hydrogen peroxide (H_2_O_2_) and malondialdehyde (MDA) and the activities of superoxide dismutase (SOD), catalase (CAT) and glutathione peroxidase (GPx) in red blood corpuscles of carp fed a diet containing an ethyl acetate extract of *Angelica sinensis* (EAE).

Treatment	Control Group	EAE Group	Control + Hypoxia–Reoxygenation	EAE + Hypoxia–Reoxygenation
Met-Hb (g L^−1^)	1.76 ± 0.09 ^c^	1.40 ± 0.09 ^a^	1.91 ± 0.12 ^d^	1.54 ± 0.11 ^b^
O_2_^−^ (U g^−1^ protein)	26.39 ± 1.36 ^a^	24.65 ± 1.05 ^a^	33.93 ± 1.17 ^c^	30.94 ± 1.62 ^b^
H_2_O_2_ (μmol g^−1^ protein)	42.37 ± 1.73 ^a^	40.30 ± 1.87 ^a^	59.92 ± 3.45 ^c^	50.07 ± 3.50 ^b^
MDA (nmol mg^−1^ protein)	2.04 ± 0.08 ^b^	1.70 ± 0.09 ^a^	2.51 ± 0.16 ^d^	2.19 ± 0.09 ^c^
SOD (U mg^−1^ protein)	76.45 ± 4.44 ^a^	80.16 ± 3.03 ^ab^	87.67 ± 3.98 ^c^	85.61 ± 5.33 ^bc^
CAT (U mg^−1^ protein)	6.16 ± 0.41 ^b^	6.97 ± 0.24 ^c^	4.83 ± 0.27 ^a^	6.07 ± 0.29 ^b^
GPx (U mg^−1^ protein)	71.43 ± 3.23 ^bc^	76.18 ± 4.88 ^c^	54.69 ± 3.09 ^a^	68.64 ± 3.28 ^b^

Values are means ± SD of 3 replicates, with 10 fish in each replicate. Values within the same line having different superscripts are significantly different (*p* < 0.05).

**Table 5:** The levels of hydrogen peroxide (H_2_O_2_) and malondialdehyde (MDA) and the activities of superoxide dismutase (SOD), catalase (CAT) and glutathione peroxidase (GPx) in branchiae of carp fed a diet containing an ethyl acetate extract of *Angelica sinensis* (EAE).

Treatment	Control Group	EAE Group	Control + Cu	EAE + Cu
H_2_O_2_ (μmol g^−1^ protein)	34.95 ± 2.78 ^a^	35.49 ± 2.43 ^a^	58.88 ± 4.03 ^c^	47.01 ± 2.90 ^b^
MDA (nmol mg^−1^ protein)	4.76 ± 0.23 ^b^	4.25 ± 0.33 ^a^	6.82 ± 0.41 ^c^	4.94 ± 0.21 ^b^
SOD (U mg^−1^ protein)	11.99 ± 0.65 ^b^	14.57 ± 0.72 ^c^	10.03 ± 0.70 ^a^	12.01 ± 0.83 ^b^
CAT (U mg^−1^ protein)	4.49 ± 0.32 ^b^	6.02 ± 0.39 ^d^	2.90 ± 0.21 ^a^	5.20 ± 0.34 ^c^
GPx (U mg^−1^ protein)	125.02 ± 9.16 ^a^	150.83 ± 9.00 ^b^	146.39 ± 9.27 ^b^	169.71 ± 12.10 ^c^

Values are means ± SD of 3 replicates, with 10 fish in each replicate. Values within the same line having different superscripts are significantly different (*p* < 0.05).

**Table 6:** The haematocrit (Hct), red blood corpuscle count (RBC), haemoglobin concentration (HbC), mean corpuscular volume (MCV), mean corpuscular haemoglobin content (MCH) and mean corpuscular haemoglobin concentration (MCHC) in blood of carp fed diets containing an ethyl acetate extract of *Angelica sinensis* (EAE).

Treatment	Control Group	EAE Group	Control + Cu	EAE + Cu
Hct (%)	0.48 ± 0.03 ^b^	0.51 ± 0.03 ^c^	0.41 ± 0.02 ^a^	0.47 ± 0.03 ^b^
RBC (10^12^ L^−1^)	2.48 ± 0.09 ^b^	2.64 ± 0.13 ^c^	2.29 ± 0.12 ^a^	2.46 ± 0.14 ^b^
HbC (g L^−1^)	98.66 ± 3.85 ^b^	107.08 ± 4.68 ^c^	85.04 ± 4.30 ^a^	97.26 ± 4.42 ^b^
MCV (fL cell^−1^)	192.71 ± 7.22 ^ab^	195.28 ± 9.33 ^b^	180.55 ± 9.12 ^a^	192.29 ± 11.34 ^ab^
MCH (pg cell^−1^)	39.70 ± 2.32 ^b^	40.46 ± 1.64 ^b^	37.11 ± 1.07 ^a^	39.68 ± 2.42 ^b^
MCHC (g L^−1^)	205.99 ± 8.03 ^a^	207.53 ± 9.07 ^a^	205.91 ± 10.41 ^a^	206.50 ± 9.38 ^a^

Values are means ± SD of 3 replicates, with 10 fish in each replicate. Values within the same line with different superscripts are significantly different (*p* < 0.05).

**Table 7:** The levels of met-hemoglobin (Met-Hb), hydroxyl radicals (·OH) and reduced glutathione (GSH) and the activities of lactate dehydrogenase (LDH), glutamate-oxaloacetate transaminase (GOT), glutamate-pyruvate transaminase (GPT) and Na^+^,K^+^-ATPase in red blood corpuscles of carp fed diets containing an ethyl acetate extract of *Angelica sinensis* (EAE).

Treatment	Control Group	EAE Group	Control + Cu	EAE + Cu
Met-Hb (g L^−1^)	1.79 ± 0.14 ^b^	1.46 ± 0.12 ^a^	1.87 ± 0.10 ^b^	1.52 ± 0.10 ^a^
·OH (U mg^−1^ protein)	20.15 ± 0.48 ^b^	18.33 ± 0.66 ^a^	25.14 ± 0.66 ^d^	21.64 ± 0.54 ^c^
GSH (μmol g^−1^ protein)	5.51 ± 0.22 ^b^	6.62 ± 0.30 ^c^	3.93 ± 0.25 ^a^	5.39 ± 0.28 ^b^
LDH (U mg^−1^ protein)	134.05 ± 9.15 ^b^	155.97 ± 10.41 ^c^	109.10 ± 5.35 ^a^	130.19 ± 10.10 ^b^
GOT (U mg^−1^ protein)	23.73 ± 1.84 ^bc^	26.01 ± 2.06 ^c^	19.91 ± 1.48 ^a^	23.04 ± 1.61 ^b^
GPT (U mg^−1^ protein)	18.09 ± 1.08 ^b^	20.84 ± 1.06 ^c^	14.83 ± 0.80 ^a^	19.87 ± 1.60 ^c^
Na^+^,K^+^-ATPase (U mg^−1^ protein)	1.72 ± 0.07 ^b^	2.16 ± 0.16 ^c^	1.36 ± 0.08 ^a^	1.81 ± 0.10 ^b^

Values are means ± SD of 3 replicates, with 10 fish in each replicate. Values within the same line with different superscripts are significantly different (*p* < 0.05).

## Data Availability

The data used to generate the results in this manuscript can be made available if requested from the corresponding author.
